# Characterization of Goat Milk Hydrolyzed by Cell Envelope Proteinases from *Lactobacillus plantarum* LP69: Proteolytic System Optimization, Bioactivity, and Storage Stability Evaluation

**DOI:** 10.3390/molecules23061317

**Published:** 2018-05-31

**Authors:** Guowei Shu, Jie Huang, Li Chen, Ni Lei, He Chen

**Affiliations:** 1 School of Food and Biological Engineering, Shaanxi University of Science and Technology, Xi’an 710021, China; huangjie9319@gmail.com (J.H.); sombre-sky@163.com (N.L.); chenhe419@gmail.com (H.C.); 2 College of Food Engineering and Nutritional Science, Shaanxi Normal University, Xi’an 710119, China

**Keywords:** *Lactobacillus plantarum* LP69, cell-envelope proteinases, proteolytic conditions, goat milk, bioactivity and storage stability

## Abstract

Despite the widespread application of lactic acid bacterium in dairy production through its contribution to acidification, development of sensorial properties, and health-promoting effects, relatively little information is available on the cell envelope proteinases (CEPs) of *Lactobacillus plantarum*, especially on the proteolytic system and the production of bioactivity peptides. In this study, CEPs from a novel *L. plantarum* LP69 were involved in goat milk hydrolysis and generated a product with high activity that showed a degree of hydrolysis of 15.68 ± 0.74%, Angiotensin I-Converting Enzyme (ACE)-inhibitory rate of 83.25 ± 1.05%, 2,2-diphenyl-1-picrylhydrazyl (DPPH) radical scavenging rate of 64.91 ± 1.27%, and hydroxyl radical scavenging rate of 89.17 ± 1.13%. The optimized hydrolysis conditions were time of 4.5 h, temperature of 41 °C, initial pH of 8.5, and enzyme to substrate ratio (*E*/*S*) of 12% (*w*/*w*) by orthogonal experiments. Application of a stabilizer greatly promoted milk stability. A well-designed stabilizer consists of 0.05% carrageenan, 0.15% gellan gum, and 0.15% sucrose esters, which significantly raised the milk stability coefficient, R, from 70.67% to 98.57%. The storage stability of milk was evaluated during 84 days at room temperature or 4 °C. Our study depicts the contribution of CEPs from *L. plantarum* LP69 in goat milk, exploring a new way for the development of a functional milk product.

## 1. Introduction

The application of cell envelope proteinases (CEPs) has been a topic of growing interest in the dairy industry in decades, since it improves the texture and sensorial properties of dairy products [1,2]. CEPs, anchored at the lactic acid bacteria (LAB) surface, are large-sized enzymes hydrolyzing β- and αs1-casein into bioactive peptides [3]. These peptides, derived through bacterial proteolysis, exhibit great biological activity with health beneficial properties, such as antihypertensive, immunomodulatory, antioxidative, antimicrobial, and cytomodulatory effects [4,5]. Fermented milks containing *L. helveticus* have been shown to induce a blood-pressure-lowering effect due to the presence of Angiotensin I-Converting Enzyme (ACE)-inhibitory peptides derived from β-casein.

In the last decades, lactococcal CEPs have been extensively characterized, both biochemically and genetically. The lactococcal CEPs play an essential role in secondary proteolysis as an intermediate activity that converts primary, chymosin-generated peptides and promotes the efficient production of amino acids. Agyei et al. [6] identified the sub-cellular location of CEPs in *L. delbrueckii* subsp. *lactis* 313 (LDL313) and compared the effects of different extraction methods for CEPs generation. The CEPs of *L. helveticus* CRL 1062 were detected in the cell membrane fraction and achieved the highest activity at pH 6.5–7.0 and a temperature of 42 °C [7]. CEP-encoding genes have been well studied in *Streptococcus thermophilus*, *L. rhamnosus*, and *L. delbrueckii*. However, relatively little is known about the CEP characteristics in *L. plantarum* compared to those of the other LAB species.

*L. plantarum* is a typical probiotic commercially used in dairy products to modulate gastrointestinal disorders and improve immunity. Our lab found that goat milk catalyzed by CEP of *L. plantarum* LP69 contains a strong ACE-inhibitory activity, but the proteolytic conditions and other bioactivity are far from clear [8]. Previous research indicates that the proteolytic system of lactobacilli consists of the CEPs and a host of intracellular peptidases such as endopeptidases, aminopeptidases, tripeptidases, and dipeptidases [3]. The CEPs are responsible for the initial stages of casein breakdown and are normally in charge of hydrolyzing over 40% of the peptide bonds of αs_1_- and β-casein, generating oligopeptides with 4–40 amino acid residues [5,9]. Emerging fermented dairy products containing *L. plantarum* and CEPs contributed health benefits. To better understand the CEPs in *L. plantarum* LP69, we optimized the proteolysis of CEPs by orthogonal experiment and evaluated the bioactivity of hydrolysate.

Milk easily undergoes flocculation due to the presence of proteins. Polysaccharide stabilizers such as pectin [10], carrageenan [11,12], gellan gum [13], xanthan gum [14], guar gum [15], soybean-soluble polysaccharides [16], and carboxymethyl cellulose (CMC) [17] are commonly used in milk for viscosity enhancement to prevent milk protein flocculation. Considering the stabilizers’ synergistic effects, promoting stability [18], we optimized the composite stabilizers in goat milk hydrolyzed by CEPs and evaluated the storage stability at different temperatures. 

## 2. Results

### 2.1. Optimization of Proteolytic Conditions of Goat Milk Catalyzed by CEP

#### 2.1.1. Effect of Proteolytic Time on Goat Milk Catalyzed by CEP

**Figure 1:**
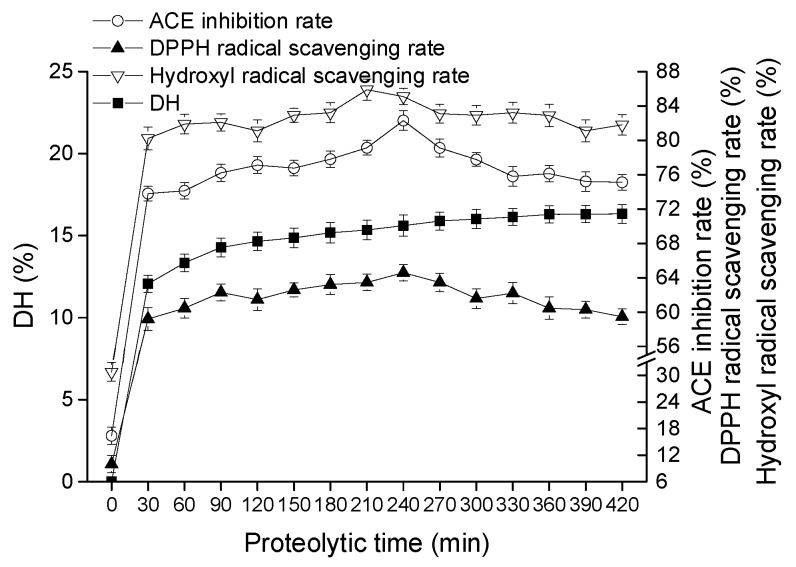
Effect of proteolytic time on DH, ACE inhibitory activity, and antioxidant activity of hydrolysate at pH of 8, temperature of 40 °C and *E*/*S* of 10% (*w*/*w*). The error bars represent standard deviation of means (*n* = 3).

Skimmed goat milk was hydrolyzed by CEP of *L. plantarum* LP69, and degree of hydrolysis (DH), Angiotensin I-Converting Enzyme (ACE) inhibition rate, 2,2-diphenyl-1-picrylhydrazyl (DPPH) radical scavenging rate, and hydroxyl radical scavenging rate were measured to evaluate the bioactivities of hydrolysate. Figure 1 indicates that the DH, ACE inhibition rate, DPPH radical scavenging rate, and hydroxyl radical scavenging rate of unfermented goat milk at initial stage were 0%, 16.37%, 9.97%, and 30.79%, respectively. DH rose with the increase in hydrolysis time, and the maximum DH rose to 15.61% at 420 min. In terms of ACE-inhibitory activity, the peak of the curve appeared at 240 min, with the highest ACE inhibition value of 82.3%. Simultaneously, DPPH radical scavenging rate also reached a maximum of 66.29%. The hydroxyl radical scavenging rate first increased and then decreased, the maximum value being 85.88% at 210 min. Therefore, in terms of generating hydrolysates with high biological activities (DPPH radical scavenging rate and hydroxyl radical scavenging), 240 min was the optimal hydrolysis time for goat milk catalyzed by CEPs. 

#### 2.1.2. Effect of Proteolytic Temperature on Goat Milk Catalyzed by CEP

**Figure 2:**
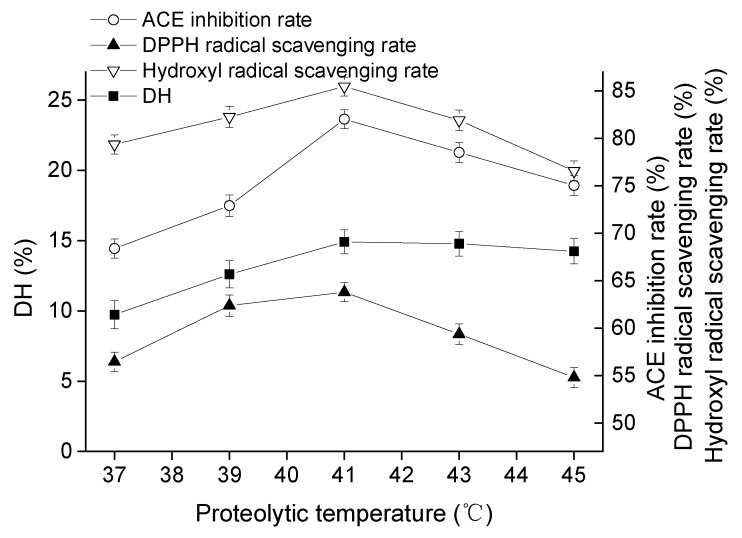
Effect of proteolytic temperature on DH, ACE inhibitory activity, and antioxidant activity of hydrolysate at pH of 8, time of 4 h, and *E*/*S* of 10% (*w*/*w*). The error bars represent standard deviation of means (*n* = 3).

The values of DH, ACE-inhibitory rate, DPPH radical scavenging rate, and hydroxyl radical scavenging rate grew similarly, the peaks reached maximum values of 14.91%, 82.03%, 63.79%, and 85.48%, respectively, at 41 °C (Figure 2). However, the growing trend of DPPH radical scavenging rate, and hydroxyl radical scavenging rate appeared to be gentle in comparison to that of DH and ACE-inhibitory rate. This could be explained by the fact that an overly high temperature might have caused proteinase denaturation and lowered enzyme activity. Thus, the optimal temperature of goat milk catalysis by CEP was 41 °C. 

#### 2.1.3. Effect of *E*/*S* on Goat Milk Catalyzed by CEP

**Figure 3:**
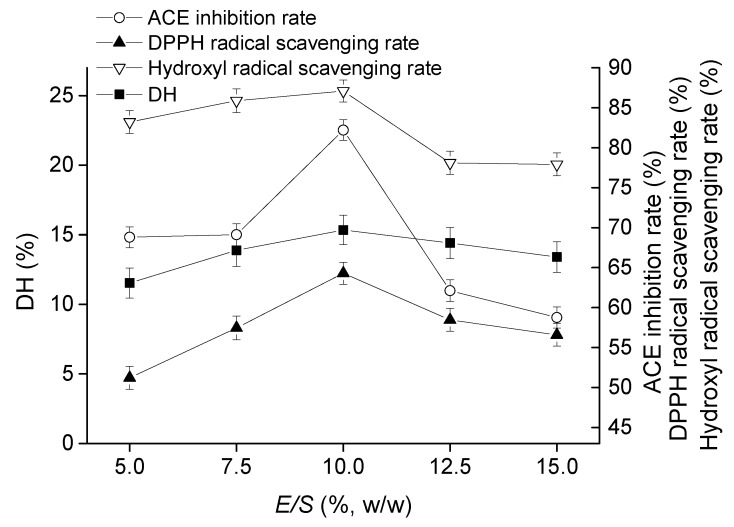
Effect of *E*/*S* on DH, ACE inhibitory activity, and antioxidant activity of hydrolysate at pH of 8, time of 4 h, and temperature of 41 °C. The error bars represent standard deviation of means (*n* = 3).

DH, ACE-inhibitory rate, DPPH radical scavenging rate and hydroxyl radical scavenging rate first increased and then decreased as *E*/*S* growing, and their values reached the maximums of 15.34%, 82.21%, 64.29%, and 87.09% when *E*/*S* was 10% (*w*/*w*) (Figure 3). Therefore, the optimal of *E*/*S* on goat milk catalyzed by CEP was *E*/*S* of 10%. 

#### 2.1.4. Effect of Initial pH on Goat Milk Catalyzed by CEP

**Figure 4:**
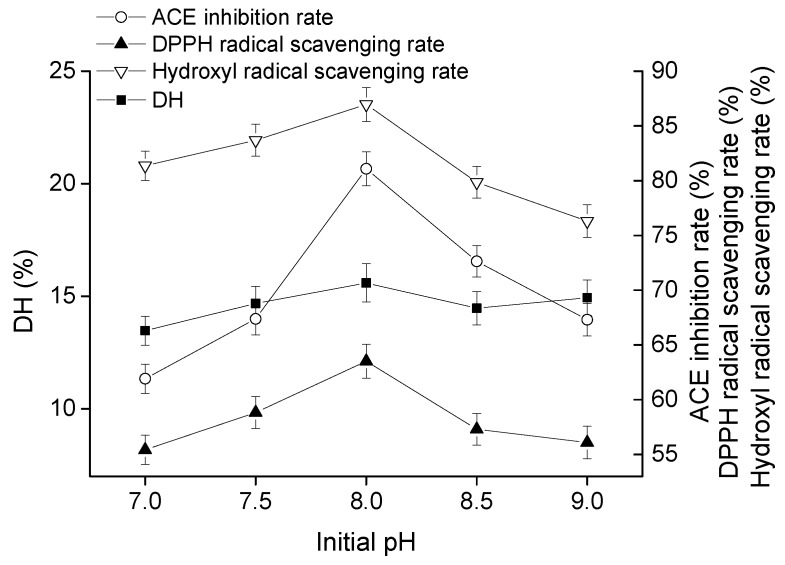
Effect of initial pH on DH, ACE inhibitory activity, and antioxidant activity of hydrolysate at time of 4 h, temperature of 41 °C, and *E*/*S* of 10% (*w*/*w*). The error bars represent standard deviation of means (*n* = 3).

The curves of DH, ACE-inhibitory rate, DPPH radical scavenging rate, and hydroxyl radical scavenging rate grew similarly. All of the peak values appeared at pH 8, each maximum values reached 15.59%, 81.09%, 63.52%, and 86.96%, respectively (Figure 4). Thus, the optimal pH was 8. 

#### 2.1.5. Optimization of the Proteolytic Conditions by Orthogonal Test

DH, ACE-inhibitory rate, DPPH radical scavenging rate, and hydroxyl radical scavenging rate were represented by Y_1_ (%), Y_2_ (%), Y_3_ (%), and Y_4_ (%), respectively, in orthogonal experiments. The design and results of orthogonal experiments are shown in Table 1, in which A is time (h), B is temperature (°C), C is *E*/*S* (%, *w*/*w*), and D is the initial pH.

According to G values in Table 1, the factors affect the DH in the order of pH, time, temperature, and *E*/*S*. The best combination of factors was A_2_B_2_C_1_D_1_ based on K value. In terms of ACE-inhibitory rate, the optimized hydrolysis condition was A_3_B_3_C_2_D_2_ while A_3_B_2_C_3_D_3_ was the most appropriate group for DPPH radical scavenging rate and hydroxyl radical scavenging rate.

The factor A_3_ was the best level on Y_2_, Y_3_ and Y_4_ but A_2_ was the best level of Y_1_; thus, choosing A_3_ served as the best time level. In addition, factor B_2_ was the best level on Y_1_, Y_3_ and Y_4_ but B_3_ was the best level of Y_2_, so B_2_ was chosen as the optimal temperature level. C_1_D_1_ and C_1_D_2_ was the optimal factor level for factors C and D when Y_1_ and Y_2_ were used as indicators. However, the best level was C_3_D_3_ when Y_3_ and Y_4_ were indicators, so the best level of C_3_D_3_ served as the optimal *E*/*S* and initial pH level. Therefore, the best combination of this test was A_3_B_2_C_3_D_3_, that is, a time of 4.5 h, a temperature of 41 °C, *E*/*S* of 12% (*w*/*w*), and initial pH of 8.5. Under these optimal conditions, the Y_1_ was 15.68 ± 0.74%, Y_2_ was 83.25 ± 1.05%, Y_3_ was 64.91 ± 1.27%, and Y_4_ was 89.17 ± 1.13%.

### 2.2. Optimization of Stabilizer of Goat Milk Catalyzed by CEPs

**Figure 5:**
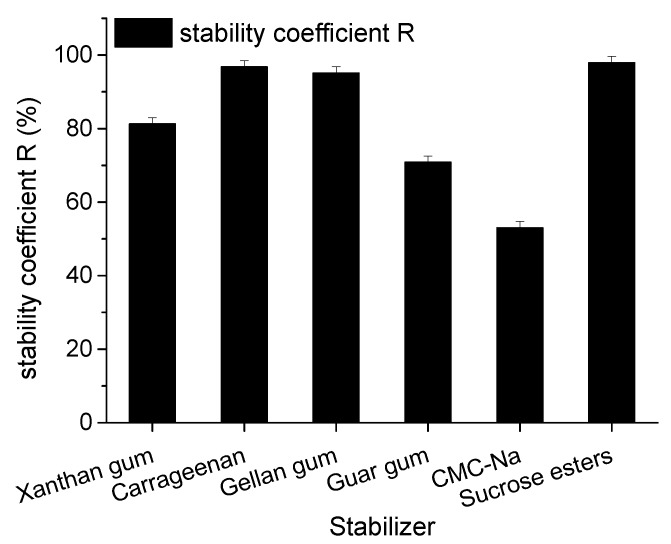
Effect of different stabilizers on stability coefficient R of goat milk catalyzed by CEP of *L. plantarum* LP69. The error bars represent standard deviation of means (*n* = 3).

Stabilizers of goat milk beverage catalyzed by CEP of *L. plantarum* LP69 were screened by measuring stability coefficient R. Figure 5 compares the six stabilizers in goat milk. Sucrose esters, carrageenan, and gellan gum were the top three candidates with corresponding stability coefficient R of 97.95%, 96.8%, and 95.12%, respectively. On the basis of a single stabilizer, the optimal three selected stabilizers were assembled by orthogonal design, and the best combination was determined by the stability coefficient R. The results are shown in Table 2.

Table 2 shows that carrageenan was most efficient to stability according to G, followed by gellan gum, and finally sucrose esters. Depending on K value, the best combination of stabilizers was A_1_B_3_C_3_, that is, carrageenan of 0.05%, gellan gum of 0.15%, and sucrose esters of 0.15%. 

### 2.3. Storage Stability of Goat Milk Catalyzed by CEP at 4 °C and Room Temperature (RT)

**Figure 6:**
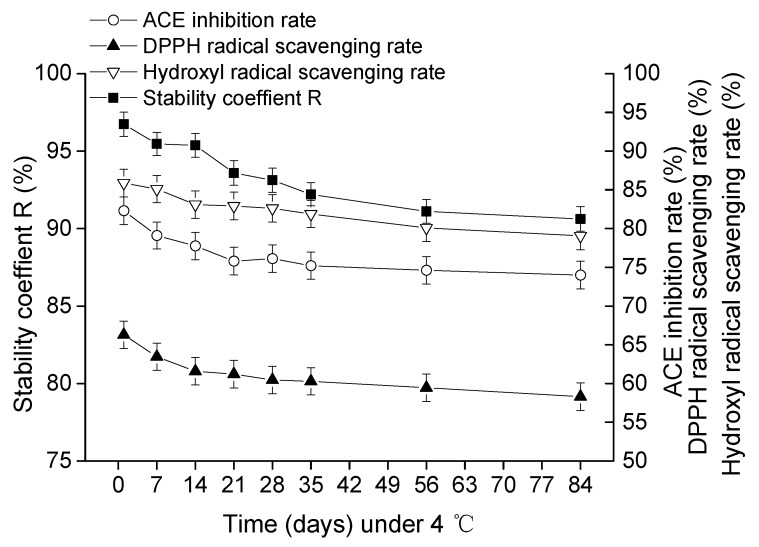
The storage stability, ACE-inhibitory activity, and antioxidant activity in goat milk catalyzed by CEP of *L. plantarum* LP69 at 4 °C. The error bars represent standard deviation of means (*n* = 3).

**Figure 7:**
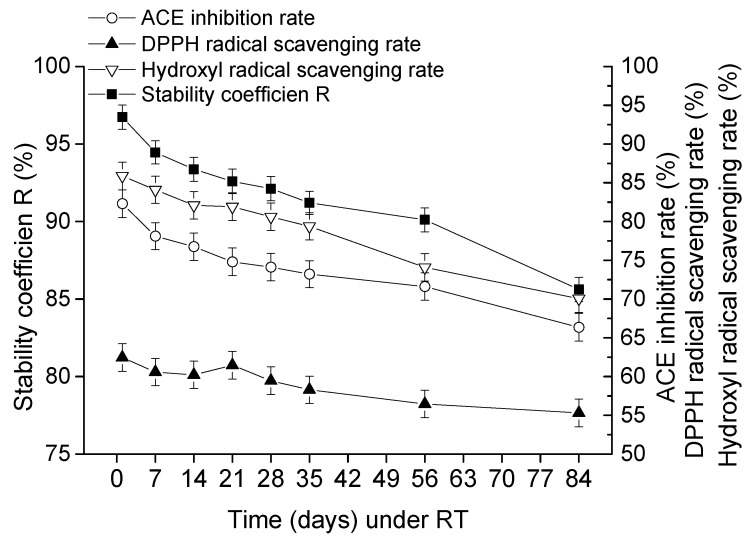
The storage stability, ACE-inhibitory activity, and antioxidant activity in goat milk catalyzed by CEP of *L. plantarum* LP69 at RT. The error bars represent standard deviation of means (*n* = 3).

The sterilized goat milk catalyzed by CEP of *L. plantarum* LP69 was stored at RT and 4 °C. The stability, ACE-inhibitory rate, DPPH radical scavenging rate and hydroxyl radical scavenging rate were investigated during the 84 days storage. Figure 6 and Figure 7 show that the stability of goat milk catalyzed by CEP at 4 °C and RT decreased from initial 96.74% to 90.62% and 85.62% during the period of storage. Under 4 °C and RT, the ACE-inhibitory rate decreased from 82.3% to 74% and 66.35%, respectively. DPPH radical scavenging rate declined from 66.29% to 58.31% and 55.31%, respectively. Hydroxyl radical scavenging rate was down from 85.88% to 79.06% and 70.06%, respectively. Taken together, the milk catalyzed by CEP of *L. plantarum* LP69 was more stable at a low temperature during storage.

## 3. Discussion

The hydrolysates produced by CEP of *L. plantarum* LP69 with ACE-inhibitory activity and antioxidant activity were applied in goat milk for nutrition promotion. Bioactive peptides were released by CEP in a multivariable-dependent process. In this study, hydrolysates of goat milk catalyzed by CEP were evaluated on temperature, time, pH, and *E*/*S* to obtain a high bioactivity product. *L. delbrueckii* subsp. *lactis* ACA-DC 178, isolated from Greek Kasseri cheese, produced a cell-wall-bound proteinase and the highest activity of crude proteinase extract at pH of 6.0 and 40 °C [19]. Pan et al. [20] determined the ACE-inhibitory activity of skimmed milk hydrolyzed by a cell-free extract of *L. helveticus* JCM1004, and optimized the proteolytic conditions at pH 6.5–7.0 and hydrolysis time of 6–10 h. Different hydrolysis conditions indicated that the optimal activities of hydrolysates produced by CEPs were strain-dependent. Our results (Figure 1, Figure 2, Figure 3 and Figure 4) show that hydrolysates from goat milk treated by CEP have strong ACE-inhibitory activity and antioxidant activity, suggesting that CEPs of *L. plantarum* have the ability to release bioactive peptides from goat milk. ACE inhibition rate (the most variable) changes from 73% to 82% with changes in proteolysis time, 68–82% with changes in temperature, 58–82% with changes in *E*/*S*, and 61–81% with changes in pH. However, the DH changes only slightly with these conditions: by 12–15%, 9–14%, 11–15%, and 13–15%, respectively, suggesting that there is no positive correlation between ACE-inhibitory activity and DH. High DH may result in further degradation of ACE-inhibitory peptides into short peptides with less activity or none [21]. Amino acid analysis of hydrolyzed fragments would help us better understand the CEPs mechanism in the future. In this study, the optimal hydrolysis conditions of goat milk were a hydrolysis temperature of 41 °C, a time of 4.5 h, a pH of 8.5, and *E*/*S* of 12%. Under these optimal conditions, the DH was 15.68 ± 0.74%, the ACE-inhibitory rate was 83.25 ± 1.05%, the DPPH radical scavenging rate was 64.91 ± 1.27%, and the hydroxyl radical scavenging rate was 89.17 ± 1.13%, which were increased by 5.16%, 2.66%, 2.19%, and 4.79%, respectively, compared to those without optimization. These indicated that ACE-inhibitory activity and antioxidant activity from goat milk catalyzed by CEP had the best efficacy under optimized conditions.

Milk beverages are usually manufactured by direct acidification or fermentation of milk with lactic acid bacteria. The pH values of these beverages are in the range of 3.4 to 4.6 [16]. However, casein micelles will aggregate and precipitate in this pH range, mostly due to the decrease in steric repulsive interactions after the collapse of the extended conformation of κ-casein, mainly on the casein micelle surface [22]. Thus, milk beverages need the addition of a stabilizer to prevent the flocculation of milk proteins. Commonly used stabilizers are sodium carboxyl methyl cellulose (CMC-Na), propylene glycol alginate (PGA), sucrose esters, xanthan gum, guar gum, pectin, and carrageenan. The mechanism of these stabilizers is that the stabilizer adsorbs onto the casein micelles by electrosorption and the adsorbed stabilizer layer on the surface of casein prevents the aggregation and sedimentation of casein micelles by electrostatic and steric stabilization. On the other hand, a non-adsorbed stabilizer can slow down the sedimentation of casein particles by increasing the viscosity of the continuous phase [17]. In this study, carrageenan, gellan gum, and sucrose esters were appropriate stabilizers and their stability coefficient, R, reached 96.8%, 95.12%, and 97.95%, respectively. Better yet, the stability coefficient R of goat milk catalyzed by CEPs from *L. plantarum* LP69 increased significantly from 70.67% to 98.57% using the composite stabilizers. The flocculation could be inhibited by the addition of more polysaccharides. The mechanism of carrageenan stabilization to milk beverages is due to the adsorption of carrageenan to casein particles, and then the negatively-charged carrageenan–casein complex being dispersed by electrostatic repulsive interaction [23]. The use of gellan gum was also beneficial for electrostatic interactions between proteins and ploysaccharides. Furthermore, sucrose esters are non-ionic surfactants containing sucrose as the hydrophilic group and fatty acids as the lipophilic group. On application of skimmed milk, sucrose esters seem more likely to bind directly to the protein through interaction of the lipophilic moiety with hydrophobic surface patches or by insertion of the alkyl chains into hydrophobic regions, so as to stabilize the protein structure [24]. To sum up, the best stabilization effect on goat milk catalyzed by CEPs was achieved with the synergistic effect of carrageenan, gellan gum, and sucrose esters.

The longevity of probiotics’ cell viability depends on the storage temperature [8]. Microorganisms tend to survive better at a relatively low temperature [25]. High temperatures generate frequent water transmission and intensive oxidation effects, which are unsuitable for bacteria survival. Measures should be taken to avoid unnecessary contact with oxygen and moisture to maintain the viability of microorganisms. With the temperature increasing, intracellular water tends to be more active and migrates more frequently, leading to a loss of viability [26]. Figure 6 and Figure 7 show that the stability coefficient R of goat milk decreased faster when stored at RT than at 4 °C. Damage to the cell membrane arose with the frequent motion of water, leading to a lower stability coefficient, R, at RT. Moreover, microorganisms generally multiply rapidly at RT. A study found that the populations of *Staphylococcus aureus*, *Aerococcus viridans*, *Acinetobacter calcoaceticus*, *Corynebacterium variabile*, and *Streptococcus uberis* were stable during seven days of storage at 4 °C [27]. During storage, milk underwent very slight lipolysis, which may be due to the enzymatic activities being hindered by a low moisture content. Fat oxidation was notable during milk storage and was enhanced by higher storage temperatures. Fat oxidation during storage at low temperatures was reported to be much less intense, and losses of fatty acid contents were almost negligible [28]. In this study, the stability coefficient (R), ACE-inhibitory rate, DPPH radical scavenging rate, and hydroxyl radical scavenging rate decreased significantly in the first 30 days and then slowed down at 4 °C. However, the stability coefficient (R), ACE-inhibitory rate, DPPH radical scavenging rate, and hydroxyl radical scavenging rate of goat milk decreased significantly over the whole storage at RT. The stability coefficient (R) after 84 days’ storage decreased rapidly by 6.12% and 11.12% at 4 °C and RT, respectively, suggesting that a functional goat milk beverage catalyzed by CEPs of *L. plantarum* LP69 was more stable at low temperatures.

## 4. Materials and Methods

### 4.1. Materials and Chemicals

Skimmed goat milk powder was obtained from Anchor, Fonterra Co-operative Group (Waikato, New Zealand). Hip-His-Leu (HHL) and ACE were purchased from Rosen Technology Co., Ltd. (Xi’an, China). Other inorganic regents were purchased from Hongyan Chemical Reagent Factory (Tianjin, China). 

### 4.2. Bacterial Strain and Media

*L. plantarum* LP69 was obtained from the School of Food and Biological Engineering, Shaanxi University of Science & Technology. The deMan, Rogosa and Sharpe (MRS) broth was obtained from Land Bridge Technology Co., Ltd. (Beijing, China) and autoclaved at 121 °C for 15 min.

### 4.3. Preparation of Cell Suspension

The *L. plantarum* LP69 freeze-dried powder was dissolved by adding MRS broth, and then *L. plantarum* LP69 was continuously activated for three generations, which inoculated 5% (*v*/*v*) into MRS broth. Inoculated MRS broth was incubated at 37 °C for 22 h. The sediments of *L. plantarum* LP69 were washed three times with 50 mM Tris-HCL buffer solution (pH 7.8) (Sigma, St. Louis, MO, USA) containing 30 mM CaCl_2_ and collected by centrifuging at 4500 r/min for 15 min at 4 °C(Model GL21, Instrumentation, Hunan). Washed cells were resuspended in 50 mM Tris-HCl buffer (pH 7.0) containing 5 mM EDTA-Na_2_ and incubated at 37 °C for 1 h. The supernatant obtained after centrifugation (4500 r/min for 15 min at 4 °C) was designated the crude CEPs.

### 4.4. Proteinase Activity Assay

Proteinase assay was determined according to the method of Folin [29]. 1 mL CEPs was added to 1 mL of substrate solution (2 mg/mL casein in 50 mM sodium phosphate buffer; pH 7.0) and incubated (40 °C 10 min). The reaction was stopped by adding 2 mL of 65.4 g/L trichloroacetic acid (TCA), holding for 20 min at 40 °C followed by centrifugation (4500 r/min, 5 min). One milliliter of supernatant was carefully taken, to which was added 5 mL of 42.4 g/L Na_2_CO_3_ followed by 1 mL of 2 N Folin & Ciocalteu’s Reagent. After incubating (40 °C 20 min) and allowing cooling to RT the absorbance at 680 nm was measured. One enzyme unit is defined as hydrolyzing casein to produce color equivalent to 1.0 μmol (181 μg) of tyrosine per hour at pH 7.0 at 35 °C The blank control replaced the enzyme solution with distilled water. The enzyme activity of crude CEPs was assayed of 26.94 U/mL.

### 4.5. Optimization of Proteolytic Conditions of Goat Milk Catalyzed by CEP

CEPs were inoculated into reconstituted skim goat milk pasteurized with 10% inoculum and fermented at 37 °C for 12 h. Aliquots of the fermented goat milk were collected and vigorously stirred. The pH of the fermented milk was adjusted to 3.4–3.6 with 1 M HCl, and the fermented milk was centrifuged at 5000 r/min for 15 min. The pH of the supernatant collected was adjusted to 8.3 with 1 M NaOH, and the supernatant was collected by centrifuging 5000 r/min for 15 min to obtain the corresponding whey fractions. The supernatants collected were filtered through a Xinhua filter and used to determine their DH, ACE-inhibitory rate, DPPH radical scavenging rate, and hydroxyl radical scavenging rate. In the first group of single factor experiment, the suspension was adjusted to various pH values from 7.0 to 9.0 for conducting a pH-dependent digestion of skimmed milk. The samples were then incubating at 41 °C for 4 h. In the second group, a time-course analysis of skimmed milk digestion was performed by incubating the suspension (pH 8) at 41 °C for various times from 0 to 420 min. In the third group, a temperature-course analysis of skimmed milk digestion was carried out by incubating the suspension (pH 8) at various temperatures from 37 to 45 °C for 4 h. In the fourth group, the proportion of CEPs and skimmed milk was from 5% to 15%. Then, the suspension was adjusted to pH 8 and incubated at 41 °C for 4 h. The fourth group of reactions was stopped by the addition of 12% TCA.

Orthogonal experiment was used to investigate the effects of enzyme to substrate ratio (*E*/*S*), temperature, pH, and time on CEPs hydrolysis. Orthogonal factor level table is shown in Table 3; A was time (h), B was temperature (°C), C was *E*/*S* (%, *w*/*w*), and D was initial pH. There are two important parameters in orthogonal experiment: *K_ji_* and *G_j_*. *K_ji_* is defined as the sum of the evaluation indexes of all levels (*i*, *i* = 1, 2, 3) in each factor (*j*, *j* = A, B, C, D). $\bar{K_{ji}}$ (mean value of *K_ji_*) was used to determine the optimal level and the optimal combination of factors. The optimal level for each factor could be obtained when $\bar{K_{ji}}$ was maximum. *G_j_* is defined as the range between the maximum and minimum value of *K_ji_* and is used for evaluating the importance of the factors, i.e., a larger *G_j_* means a greater importance of the factor. 

### 4.6. Optimization of Stabilizers of Goat Milk Catalyzed by CEP

About 0.8% β-cyclodextrin was used to remove bitterness of the goat milk beverage. Then, the effects of xanthan gum, carrageenan, gellan gum, guar gum, CMC-Na, and sucrose esters on the stability of goat milk beverage catalyzed by CEP were investigated. These stabilizers at the concentration of 0.25% (***w*/*v***) were added to 20 mL of hydrolysate solution, respectively. The stability coefficient of each sample was determined to screen the optimal stabilizer. Then, the optimal three stabilizers were designed by orthogonal design. The best compounding ratio of combination stabilizers was determined by the stability coefficient. Orthogonal factors and levels are shown in Table 4. 

### 4.7. The Storage Stability of Goat Milk Catalyzed by CEP

The prepared goat milk catalyzed by CEP of *L. plantarum* LP69 were stored at RT and 4 °C refrigerator, respectively, and the stability measured after seven, 14, 21, 28, 35, 56, and 84 days. ACE-inhibitory rate, DPPH radical scavenging rate, and hydroxyl radical scavenging rate were used as evaluation indexes to determine suitable storage conditions. 

### 4.8. Analytical Testing Methods

#### 4.8.1. Assay for ACE-Inhibitory Activity

200 μL of HHL buffer (5 mM HHL in 0.1 M borate buffer containing 0.025 M borax buffer, pH 8.3) were mixed with 80 μL of CEP solution and pre-incubated for 5 min at 37 °C. The reaction was initiated by adding 20 μL of ACE (dissolved in borate buffer, 0.1 UN/mL), and the mixture was incubated for 30 min at 37 °C. The reaction was stopped by adding 250 μL of 1.0 N HCl and mixed with 1.7 mL of ethyl acetate. The liberated hippuric acid was extracted with ethyl acetate, and 1.0 mL of ethyl acetate was removed from the ethyl acetate layer. The material was dried at 120 °C for 30 min and redissolved in 1.0 mL of distilled water. The absorbance was measured at 228 nm using a spectrophotometer to measure the ACE activity. The average value from three determinations at each concentration was used to calculate the ACE-inhibitory rate. The extent of inhibition was calculated as follows:

(1) $$\mathrm{ACE}-\mathrm{inhibitory}\;\mathrm{rate}\;(\%)=\frac{B-A}{B-C}\times 100\%,$$

where A was the absorbance in the presence of ACE and with the ACE-inhibitory component, B was the absorbance with ACE and without the ACE-inhibitory component, C was the absorbance without ACE or ACE-inhibitory component.

#### 4.8.2. Determination of DPPH Radical Scavenging Rate

Two milliliters of enzyme solution was added in 2 mL of 0.1 mM DPPH-ethanol solution (DPPH (Sigma, USA) dissolved in 95% ethanol). The mixture was shaken well before placed in the dark for 30 min and then measured the absorbance at 517 nm as A_1_. Two milliliters of enzyme solution and 95% ethanol were added in another tube, respectively. The absorbance of mixture at 517 nm as A_2_ was measured and was set as the blank group. In the control group, the absorbance value A_3_ was determined from a mixture of 2 mL of DPPH–ethanol solution and 2 mL of 95% ethanol. Equal volumes of water and ethanol were used to get to zero. The formula was as follows:

(2) $$\mathrm{DPPH}\;\mathrm{radical}\;\mathrm{scavenging}\;\mathrm{rate}=\left(1-\frac{A_{1}-A_{2}}{A_{3}}\right)\times 100\%,$$

where A_1_ was the absorbance of the sample, A_2_ was the absorbance of the blank group, and A_3_ was the absorbance of the control group.

#### 4.8.3. Determination of Hydroxyl Radical Scavenging Rate

One milliliter of the sample, 1 mL of 9 mM ferrous sulfate, and 1 mL of 10 mM hydrogen peroxide were added to the test tube. The mixture was incubated at 37 °C for 10 min and then was added to 1 mL of 9 mM salicylic acid and incubated for 10 min at 37 °C. After the reaction, the absorbance of A_510_ was determined and pure water was used as the blank control. The formula was as follows:

(3) $$\mathrm{Hydroxyl}\;\mathrm{radical}\;\mathrm{scavenging}\;\mathrm{rate}=\left(1-\frac{A_{1}}{A_{2}}\right)\times 100\%,$$

where A_1_ was the absorbance of the sample and A_2_ was the absorbance of the blank group.

#### 4.8.4. Determination of DH

The proteinase was added to substrate in water bath under the optimal temperature and pH of the protease. During the proteolytic reaction, a solution of 0.1 M NaOH was added dropwise at regular intervals to keep the pH constant. The volume of the NaOH used was recorded and then the DH of the protein was calculated according to the pH-Stat method. The formula was as follows:

(4) $$\mathrm{Degree}\;\mathrm{of}\;\mathrm{hydrolysis}\;(\mathrm{DH})=\frac{B\times \mathrm{Mb}}{\alpha \times \mathrm{Mp}\times 8.2}\times 100\%,$$

where B was the volume of the NaOH, Mb was the 0.1 M NaOH, α was the reciprocal of the DH of casein hydrolysis reaction, and Mp was the quality of protein.

#### 4.8.5. Determination of Stability of Goat Milk

The absorbance at 550 nm of the sample was measured as A_before_. Three milliliters of the sample were centrifuged at 3500 r/min for 15 min. Then 1 mL of supernatant was diluted five-fold. The absorbance at 550 nm after centrifuging was measured as A_after_. The ratio of the absorbance after centrifuging and before centrifuging was the stable coefficient R of the protein.

(5) $$\mathrm{The}\;\mathrm{stable}\;\mathrm{coefficient}\;R=\frac{A_{\mathrm{before}}}{A_{\mathrm{after}}}\times 100\%,$$

where A_before_ was the absorbance at 550 nm of the sample before centrifuging and A_after_ was the absorbance at 550 nm of the sample after centrifuging.

### 4.9. Statistical Analysis

Each experiment was carried out at least in triplicate. Statistical analysis was performed using the Origin 9 software package (Origin Lab Inc., Alexandria, VA, USA) and Microsoft Excel 2010 (Redmond, WA, USA). 

## 5. Conclusions

The combination hydrolysis conditions of goat milk catalyzed by CEP of *L. plantarum* LP69 were optimized by an orthogonal experiment and determined as: a hydrolysis time of 4.5 h, temperature of 41 °C, initial pH of 8.5, and *E*/*S* of 12% (*w*/*w*) with maximum DH of 15.68 ± 0.74%, ACE inhibition rate of 83.25 ± 1.05%, DPPH radical scavenging rate of 64.91 ± 1.27%, and hydroxyl radical scavenging rate of 89.17 ± 1.13%. The best stabilization effect on milk beverages was achieved through the synergistic effect of carrageenan, gellan gum, and sucrose esters. The composite stabilizer consists of 0.05% carrageenan, 0.15% gellan gum, and 0.15% sucrose esters. The stability coefficient R of goat milk catalyzed by CEPs from *L. plantarum* LP69 increased significantly to 98.57%. The storage stability study revealed that the bioactivity of milk catalyzed by CEP was more stable under low temperature. The stability coefficient, R, after 84 days’ storage at 4 °C and RT decreased rapidly by 6.12% and 11.12%, respectively, indicating that a functional goat milk beverage catalyzed by CEP of *L. plantarum* LP69 was more stable at a low temperature.

## Figures and Tables

**Table 1:** Analysis of orthogonal experiments for the effects of time (A), temperature (B), *E*/*S* (C), and initial pH (D) on CEPs hydrolysis. DH, ACE-inhibitory rate, DPPH radical scavenging rate, and hydroxyl radical scavenging rate were represented by Y_1_ (%), Y_2_ (%), Y_3_ (%), and Y_4_ (%), respectively.

Trial No.		Factors				Y_1_/%	Y_2_/%	Y_3_/%	Y_4_/%
		A/h	B/°C	C/%	D				
1		1(3.5)	1(40)	1(8)	1(7.5)	13.73 ± 0.39	63.85 ± 0.49	56.37 ± 1.80	85.71 ± 1.07
2		1	2(41)	2(10)	2(8.0)	12.89 ± 1.31	68.57 ± 0.68	61.92 ± 0.51	80.46 ± 0.72
3		1	3(42)	3(12)	3(8.5)	13.50 ± 0.77	74.09 ± 1.39	60.48 ± 1.50	87.04 ± 0.36
4		2(4.0)	1	2	3	14.29 ± 1.15	78.46 ± 1.07	58.57 ± 1.71	79.53 ± 0.41
5		2	2	3	1	15.61 ± 0.46	61.57 ± 0.99	62.39 ± 2.07	82.16 ± 1.14
6		2	3	1	2	14.03 ± 0.72	80.51 ± 1.03	59.02 ± 0.73	78.47 ± 1.03
7		3(4.5)	1	3	2	12.74 ± 0.22	76.06 ± 0.21	60.64 ± 0.93	86.39 ± 0.58
8		3	2	1	3	15.04 ± 0.34	71.43 ± 0.65	63.42 ± 1.39	90.62 ± 0.29
9		3	3	2	1	14.75 ± 1.55	75.02 ± 1.22	58.45 ± 1.21	84.80 ± 1.17
Y_1_/%	$\bar{K_{1}}$	13.37	13.59	14.27	14.70				
	${\bar{k}}_{2}$	14.64	14.51	13.98	13.22				
	$\bar{k_{3}}$	14.18	14.09	13.95	14.28				
	*G*	1.27	0.92	0.32	1.48				
Y_2_/%	$\bar{K_{1}}$	63.84	72.79	71.93	66.81				
	${\bar{k}}_{2}$	73.51	67.19	74.02	75.05				
	$\bar{k_{3}}$	74.17	76.54	70.57	74.66				
	*G*	10.33	9.35	3.45	8.24				
Y_3_/%	$\bar{K_{1}}$	59.59	58.53	59.6	59.07				
	${\bar{k}}_{2}$	60.00	62.58	59.64	60.53				
	$\bar{K_{3}}$	60.84	59.32	61.17	60.82				
	*G*	1.25	4.05	1.57	1.75				
Y_4_/%	$\bar{K_{1}}$	84.40	83.88	84.94	84.22				
	${\bar{k}}_{2}$	80.05	84.41	81.60	81.77				
	$\bar{k_{3}}$	87.27	83.44	85.20	85.73				
	*G*	7.22	0.97	3.60	3.96				

*G*: the range between the maximum and minimum value of $\bar{K_{i}}$; *K_i_*: the sum of the evaluation indexes of all levels (*i*, *i* = 1, 2, 3) in each factor; $\bar{K_{i}}$: mean value of *K_i_*.

**Table 2:** The analysis of orthogonal experiments for stabilizers in goat milk catalyzed by CEP of *L. plantarum* LP69.

Trial No.	Factors			Stability Coefficient R/%
	Carrageenan (*w*/*v*)/%	Gellan Gum (*w*/*v*)/%	Sucrose Esters (*w*/*v*)/%	
1	1(0.05)	1(0.05)	1(0.05)	96.75 ± 0.24
2	1	2(0.10)	2(0.10)	95.86 ± 0.47
3	1	3(0.15)	3(0.15)	98.57 ± 0.22
4	2(0.10)	1	2	91.50 ± 0.26
5	2	2	3	91.65 ± 0.30
6	2	3	1	94.03 ± 0.21
7	3(0.15)	1	3	97.52 ± 0.48
8	3	2	1	92.45 ± 0.23
9	3	3	2	96.85 ± 0.16
$\bar{K_{1}}$	97.06	93.57	94.41	
${\bar{k}}_{2}$	92.39	93.32	94.74	
$\bar{k_{3}}$	95.60	96.48	95.91	
*G*	4.67	3.16	1.50	

*G*: the range between the maximum and minimum value of *K_i_*; *K_i_*: the sum of the evaluation indexes of all levels (*i*, *i* = 1, 2, 3) in each factor; $\bar{K_{i}}$: mean value of *K_i_*.

**Table 3:** The factor and levels of orthogonal experiment for proteolytic conditions of goat milk catalyzed by CEP of *L. plantarum* LP69.

Level	Factors			
	Time/h, (A)	Temperature/°C, (B)	*E*/*S* (*w*/*w*)/%, (C)	Initial pH, (D)
1	3.5	40	8	7.5
2	4.0	41	10	8.0
3	4.5	42	12	8.5

**Table 4:** The factor and level of orthogonal experiment for stabilizer of goat milk catalyzed by CEP of *L. plantarum* LP69.

Level	Factors		
	Carrageenan (*w*/*v*)/%	Gellan Gum (*w*/*v*)/%	Sucrose Esters (*w*/*v*)/%
1	0.05	0.05	0.05
2	0.10	0.10	0.10
3	0.15	0.15	0.15
